# HIV-1 Tat protein binds to TLR4-MD2 and signals to induce TNF-α and IL-10

**DOI:** 10.1186/1742-4690-10-123

**Published:** 2013-10-28

**Authors:** Nawal Ben Haij, Kaoutar Leghmari, Rémi Planès, Nathalie Thieblemont, Elmostafa Bahraoui

**Affiliations:** 1 Université Paul Sabatier, EA 3038, 118 Route de Narbonne, 31062 Toulouse, France; 2 INSERM, U1043, CPTP, CHU purpan, BP3028, 31024 Toulouse, Cedex3, France; 3 CNRS, U5282, CPTP, CHU purpan, BP3028, 31024 Toulouse, Cedex3, France; 4 CNRS UMR 8147, Necker Hospital, Paris, France; 5 Faculté de Medicine, Necker Hospital, Paris Descartes University, Paris, France

**Keywords:** HIV-1, TLR4, Tat, IL-10, TNF-α

## Abstract

**Background:**

HIV-1 infection results in hyper-immune activation and immunological disorders as early as the asymptomatic stage. Here, we hypothesized that during early HIV-1 infection, HIV-1 Tat protein acts on monocytes/macrophages to induce anti-inflammatory and proinflammatory cytokines and participates in immune dysregulation.

**Results:**

In this work we showed that Tat protein: i) by its N-terminal domain induces production of both IL-10 and TNF-α in a TLR4-MD2 dependent manner, ii) interacts specifically with TLR4-MD2 and MD2 with high affinity but not with CD14, iii) induces *in vivo* TNF-α and IL-10 in a TLR4 dependent manner.

**Conclusions:**

Collectively, our data showed for the first time that, HIV-1 Tat interacts physically with high affinity with TLR4-MD2 to promote proinflammatory cytokines (TNF-α) and the immunosuppressive cytokine IL-10 both involved in immune dysregulation during early HIV-1 infection and AIDS progression.

## Background

HIV-1 infects numerous cells of the immune system, essentially CD4 T-cells, monocytes/macrophages and, to a lesser extent, dendritic cells [1] leading to the establishment of a persistent chronic hyper-immune activation [2]. As consequence, this abnormal hyperstimulation inevitably leads to the weakening of the immune system that facilitates HIV-1 replication, virus persistence and AIDS disease progression [2-4]. HIV-1 immune activation is associated with the production of several cytokines, including TNF-α, a pro-inflammatory cytokine, and IL-10, a highly immunosuppressive cytokine, two cytokines that have been involved in the immune dysregulation observed in HIV-1 infected patients [5]. Indeed, HIV-1 is able to activate cells of the innate immune system via various pathways. By its nucleic acids, HIV-1 activates Toll-like receptor 7/8 (TLR7/8) and TLR3 to activate innate signaling of HIV-1 infected cells and to induce proinflammatory cytokines, including TNF-α and type I IFNs, that contribute to immune activation and viral replication [6-8]. At least 10 TLR have been reported in humans and 13 in mice [9], expressed by the cells of the immune system. Like TLR, other innate immune pattern recognition receptors (PRR), that play an essential role in the initiation of the innate and adaptive immune responses, including NOD-like receptors (NLR), RIG-like receptors (RLR) and C-type lectin receptors (CLR), also recognize conserved pathogen-associated molecular patterns (PAMP) to activate proinflammatory cytokines and chemokines [9].

Our group [10-13] and others [14,15] have shown that HIV-1 Tat protein is able to stimulate proinflammatory (IL1-β, IL-6, IFN-γ, TNF-α) and anti-inflammatory (IL-10) cytokines in human monocytes/macrophages. Tat protein is an 86 to 104 amino-acids polypeptide of 14 kDa, known for its crucial transactivation activity of HIV-LTR [16]. Tat protein is structured in several domains including the N-terminal region 1–47 and the basic region, which is essential for Tat internalization, nuclear localization and RNA binding at the LTR-TAR region [17]. Tat protein is found at nM levels in the serum of HIV-1 infected patients [18-20]. However, taking into account that a fraction of Tat protein remained adsorbed on the cell surfaces, the determined soluble Tat concentration is probably underestimated and could be much larger near the lymphoïd organs and in the vicinity of infected cells [21].

Beside its crucial role in activating viral replication, Tat also participates in the pathogenesis of HIV-1 infection by its capacity to interact with infected or not infected cells [22]. Tat also, contributes to the spread of HIV-1 through its effect on the increase of CCR5 and CXCR4 surface expression [23]. Tat has been found to induce neurotoxicity in the central nervous system [24,25] and apoptosis in CD4 T-cells [26]. Moreover, several studies suggest a direct effect of Tat protein in the structural and immunological dysfunctions observed early after infection, in the gastrointestinal tract (GALT) from HIV-1 infected patients [2]. Indeed, it was reported that Tat protein can act directly on the GALT, by impairing intestinal glucose absorption [27] or indirectly by boosting abnormaly immune activation, which is exacerbated later following the breakdown of the mucosal barrier and the translocation of the bacterial product into the blood [2].

While some of Tat effects are mediated after intracellular uptake of Tat, others are mediated by the interaction of extracellular Tat with cellular receptors. Different domains of Tat have been implicated in interactions with membrane receptors: the N-terminal region with CD26 receptor [28] and L-Type calcium channel [29] the tripeptide RGD with integrin α_v_β_3_ and α_5_β_1_ of dendritic cells [30], and the basic domain with membrane lipids [25] or with the Flk-1/KDR receptor [31]. Among these potential Tat receptors, it would be of importance to determine which receptor(s) participate to the activation of signalling pathways that lead to the production of proinflammatory and anti-inflammatory cytokines, reported by our group and others, which seem to be strongly involved in the abnormal immune activation and immune dysregulation.

In this study, we advance TLR4 as a potential candidate receptor for Tat protein for the following reasons: i) Tat protein induces the production of TNF-α and IL-10 by human monocytes/macrophages by acting at the cell membrane level, ii) TLR4 is expressed on the surface of monocytes/macrophages, iii) the signalling pathways activated by Tat, including MAPkinases, PKC and NF-κB [11] are also activated following the engagement of the TLR4 pathways [32].

Our results presented in this study, showed that Tat protein induced TNF-α and IL-10 production in monocytes-macrophages from human and mice cells but not in macrophages from TLR4 KO mice. Further we showed that Tat protein by its N-terminal domain 1–45 interacts with high affinity with TLR4-MD2 receptor on human monocyte-macrophage cells to induce TNF-α and IL-10, two cytokines implicated in the hyperactivation and dysregulation of the immune system in HIV-1 infected patients.

## Results

### Tat protein induces the production of TNF-α and IL-10 by acting at cell membrane level in human monocytes

Tat protein contains a nuclear localization sequence between amino acids 49 and 57 which allows it to be taken up by cells into the nucleus. Thus, Tat protein could act at either the membrane and/or the nucleus level. Previously, we showed that stimulation of human monocytes with synthetic or recombinant proteins, GST-Tat 1-101 or GST-Tat 1–45, but not GST-Tat 30–72 or GST alone, activated the production of TNF-α and IL-10 [11].

In addition, we previously showed that Tat oxidation by H_2_O_2_, its trypsin digestion or heating (5 min at 95°C) totally abolished the capacity of Tat to induce the production of TNF-α and IL-10, while such treatments had no effect on the capacity of LPS to stimulate the production of these cytokines [11]. Using the LAL assay, we showed that the Tat protein used in this work contained no endotoxins within the limit of detection of the test (less than 50 pg/ml). Likewise, LPS at 50 pg/ml did not cause the production of TNF-α and IL-10 in our experiments. All these characterizations exclude contamination with endotoxins and indicate the direct implication of Tat protein, by acting at the cell membrane surface, by its N-terminal domain to induce the TNF-α and IL-10 production.

### Tat protein induces TLR4-dependent cytokine production in human monocytes

**Figure 1:**
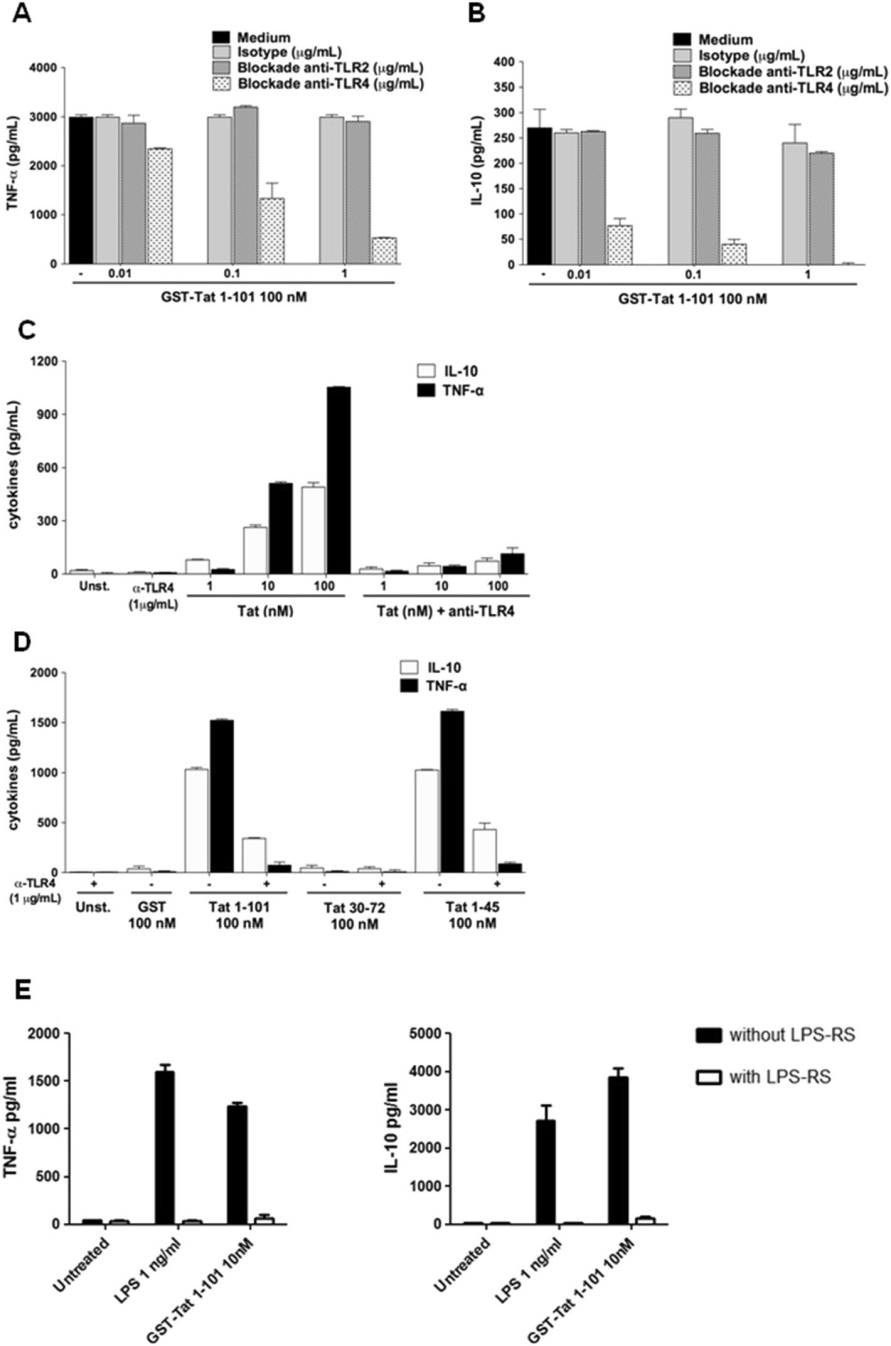
**HIV-1 Tat-protein-induced TNF-α and IL-10 is TLR4-dependent. A-B)** Monocytes were pretreated or not with increasing amounts of blocking antibodies against TLR4 or TLR2 or isotype control for 1h before stimulation by GST-Tat 1–101. **C)** Mab anti-TLR4 (1 μg/ml) were incubated for 1 h with 10^6^ human monocytes before stimulation with 1, 10 or 100 nM of Tat or with, **D)** 100 nM of GST-Tat 1–101, GST-Tat 1–45, or GST-Tat 30–72. GST was used as a control. After 24 h, TNF-α and IL-10 in the supernatant (SN) were measured by ELISA. After 24 h, TNF-α and IL-10 were measured in the SN. The values are representative of three independent experiments. **E)** Monocytes were treated with LPS (1 ng/ml) or GST-Tat1-101 (10 nM) in the presence or not of LPS-RS (1 μg/ml). After 24 h, TNF-α and IL-10 were quantified in the culture supernatant by ELISA. Data represent means +/− SD.

To investigate the role of TLR4-MD2 as a potential receptor implicated in the production of TNF-α and IL-10 by Tat, we evaluated the inhibitory effect of the anti-TLR4 blocking monoclonal antibody (Mab), clone HTA125, on Tat-induced cytokine production. To this end, primary human monocytes were pretreated with increasing amounts of anti-TLR4 Mab (0.01–1 μg/ml) before stimulation by Tat. In these conditions anti-TLR4 antibodies inhibited Tat-induced cytokine in a dose dependent manner (Figure 1A-B). Total inhibition was obtained with anti-TLR4 Mab at 1 μg/ml. Similarly, when monocytes were pretreated with saturating amount of anti-TLR4 antibodies (1μg/ml) and then stimulated with increasing concentrations of Tat 1–101 (1–100 nM) or its deleted mutants Tat 1–45, strong inhibition of TNF-α and IL-10 were observed (Figure 1C-D). No inhibition was observed when Tat stimulation was performed in the presence of anti-TLR2 or with irrelevant IgG antibodies harbouring the same isotype as HTA125 Mab in control experiments (Figure 1A). More interestingly, we showed that LPS-RS (from *Rhodobacter Sphaeroides*), previously described as a potent antagonist of LPS [33], is also able to block the ability of Tat to induce TNF-α and IL-10 production (Figure 1E). Finally, as expected, we showed that HTA125 Mab also totally inhibited LPS-induced cytokine production (Additional file 1: Figure S2).

Altogether, these results indicate that Tat induces TLR4-dependent production of IL-10 and TNF-α in human monocytes.

### HIV-1 Tat protein interacts physically with TLR4-MD2

Taking these data into consideration, we investigated the capacity of Tat to interact directly with TLR4 and its cofactors MD2 and CD14. MD2 is a soluble glycosylated polypeptide of 160 amino acids which associates with high affinity to the ectodomain of TLR4, while CD14 is a glycosylphosphatidylinositol (GPI) membrane glycoprotein of 375 amino-acids which seems to play an important role in the trafficking of TLR4 and other receptors, including TLR3, TLR7 and TLR9 [34].

**Figure 2:**
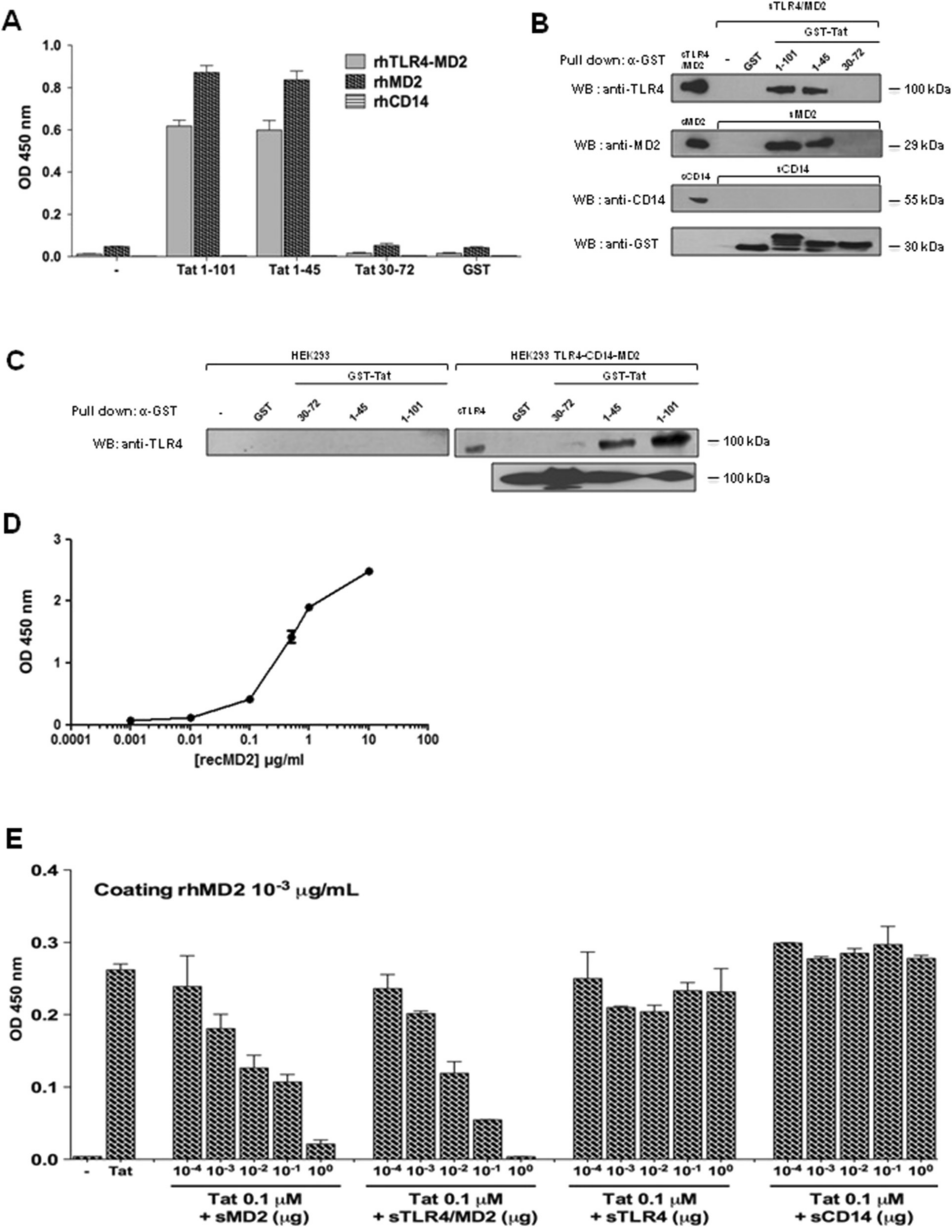
**Tat protein and its N-terminal fragment Tat 1–45 interact physically with TLR4-MD2 and MD2 but not with CD14. A)** rh TLR4-MD2, MD2 and CD14 were coated at 1 μg/ml in the wells. After incubation with GST-Tat 1–101, GST-Tat 1–45, GST-Tat 30–72 or GST control (1 μM), interaction of Tat with coated rh proteins was analyzed by ELISA. The data represent OD at 450 nm and are representative of one of three independent experiments. **B-C)** GST-pull down experiments: GST-Tat 1–101, GST-Tat 1–45, GST-Tat 30–72 or GST control (1 μM) coupled to glutathione-agarose beads were incubated **B)** with recombinant TLR4-MD2, MD2 and CD14 (1 μg/ml) or **C)** with 500 μg of cellular extracts from HEK cell expressing TLR4-MD2-CD14 or not. After washes, retained and unretained proteins fractions were evaluated by SDS-PAGE and western blot using antibodies directed against CD14, MD2, TLR4 and GST. **D)** Evaluation of the capacity of recombinant TLR4 to interact with recombinant MD2. rh TLR4 was coated at 1 μg/ml in the wells. After incubation with various concentrations of MD2 (1-10 μg/ml). TLR4-MD2 interaction was analyzed by ELISA using anti-MD2 monoclonal antibodies. **E)** rhMD2 and rhTLR4-MD2 compete for Tat-rhMD2 interaction: GST-Tat 1–101 (0.1 μM) were pre-incubated for 1 h with PBS (control) or with increasing amounts of soluble rhMD2, rhTLR4-MD2, rhTLR4 or rhCD14 before incubation with the coated rhMD2. Binding of Tat to rhMD2 was analyzed as described above by measuring OD at 450 nm. The data represent OD at 450 nm and are representative of one of three independent experiments.

**Figure 3:**
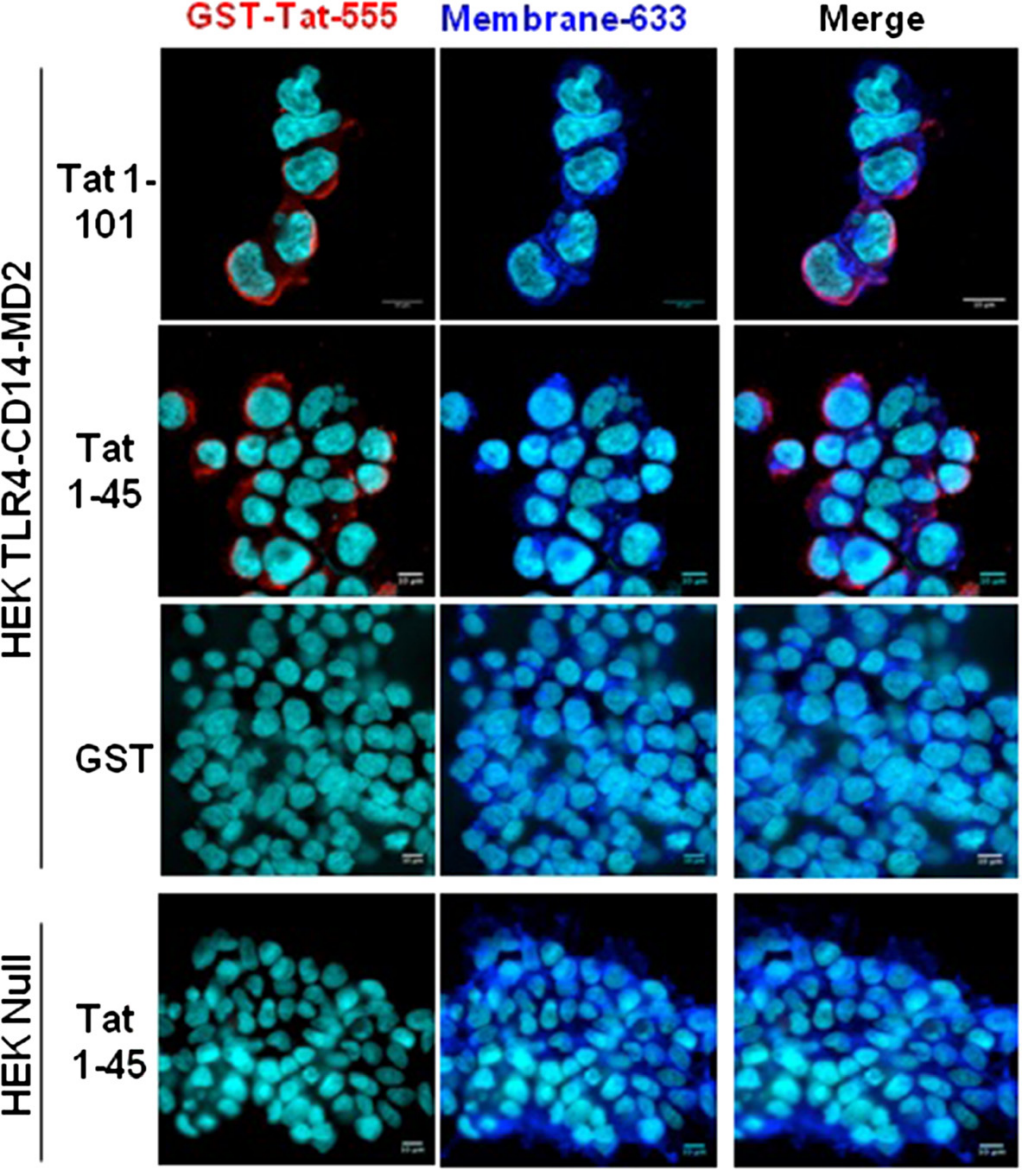
**Analysis of Tat and- TLR4-MD2 labelling at the cell surface.** HEK cells null or HEK-TLR4-MD2-CD14 were pre-incubated with GST-Tat 1–101 or GST-Tat 1–45 or control GST during 15 min. GST+/−Tat were labelled with an anti-GST antibodies (red). WGA-633 (blue) was used to label the membrane of cells and DAPI (cyan) was used as a nuclear marker. Scale bars are represented right down of each images.

To investigate whether Tat was able to interact physically with TLR4-MD2 complex, MD2 or CD14 recombinant proteins were tested for their capacities to interact, in a solid phase assay, with HIV-1 Tat protein or its deleted mutants Tat 1–45 and Tat 30–72. The results depicted in Figure 2A show a direct interaction of Tat with TLR4-MD2 or with MD2 alone. In contrast, no interaction was observed between Tat and CD14 (Figure 2A). As control, when GST was used, no binding with TLR4-MD2 or MD2 was detected (Figure 2A). To identify the domain of Tat implicated in this interaction, the N-terminal domain Tat 1–45 and the central domain, Tat 30–72, were tested in the same assays. The results showed that the N-terminal domain, Tat 1–45, as Tat 1–101, also, interacted strongly with MD2 and TLR4-MD2, but not with CD14 (Figure 2A). In contrast, no binding was observed with the Tat 30–72 fragment or with GST control (Figure 2A). In a parallel assay, GST-Tat 1–101, GST-Tat 1–45, GST-Tat 30–72 and GST previously coupled to glutathion-agarose beads, were tested for their capacity to interact with soluble recombinant TLR4-MD2, MD2 or CD14. After incubation and washes, the preformed complexes were analyzed by SDS-PAGE and western blot. The corresponding results, shown in Figure 2B, clearly confirmed the capacity of Tat to interact, *via* its N-terminal fragment, with TLR4-MD2 and MD2, but not with CD14. In line with the binding assay data, similar results were obtained when the same experiments were performed using, as source of TLR4/MD2/CD14 cell lysate proteins prepared from HEK293 cells stably transfected with TLR4-CD14-MD2 (Figure 2C). Non-transfected HEK cells were used as controls (Figure 3C). In addition, recombinant GST-Tat proteins (Additional file 2: Figure S1A) and recombinant TLR4, MD2 and CD14 (Additional file 2: Figure S1C) were characterized by SDS-PAGE. Further we have evaluated the native-like conformations of TLR4 and MD2 by demonstrating, in a binding assay, their capacity to interact physically and in a dose dependent manner (Figure 2D).

In order to demonstrate the specificity of Tat-TLR4-MD2 interactions, Tat-MD2 or Tat-TLR4-MD2 interactions were further analyzed in a molecular binding assay. Binding was performed in the presence of various concentrations of MD2, TLR4-MD2 or Tat. The results in (Additional file 3: Figure S3A-B), show that Tat (1 μM) binds to MD2 (1 pg/mL to 1μg/mL) in a dose dependent manner, with a clear saturation plateau. Similarly, the binding of MD2 (10 ng/mL) to increasing amounts of Tat (10^-11^ to 10^-6^ M) showed that the formation of Tat-MD2 or Tat-TLR4-MD2 complexes were dependent on the Tat concentrations, with a saturation plateau at 10^-6^ M of Tat (Additional file 3: Figure S3C-D).

The specificity of Tat-MD2 and Tat-TLR4-MD2 interactions were further characterized by testing the capacity of soluble MD2, TLR4-MD2, TLR4 or CD14 to compete for these interactions. The results depicted in Figure 2E, clearly show the capacity of soluble MD2 (100 pg - 1 μg) to inhibit the binding of Tat to coated MD2 in a dose dependent manner (Figure 2E). Strong inhibition (more than 95%) was obtained with soluble MD2 used at 1 μg/ml. The concentration of soluble MD2 (K_0.5_) capable of inhibiting Tat-MD2 interaction by 50% was about 4.10^-9^ M. This value of K_0.5_, which can be considered as an apparent dissociation constant, indicates that Tat recognizes MD2 with a relatively high affinity. In agreement with the direct binding data, soluble TLR4-MD2 is also able to totally inhibit Tat-MD2 or Tat-TLR4-MD2 interactions when used at 1 μM (Figure 2E). The K_0.5_ of TLR4-MD2, about 10^-9^ M, is 2.5 times less than that obtained with MD2 alone, suggesting a higher affinity of MD2, for Tat, when it is associated with TLR4. In contrast, when soluble TLR4 or CD14 were used as competitors, no significant inhibitions were observed (Figure 2E). Similar results were obtained when soluble TLR4-MD2, TLR4, or CD14 were used to compete for MD2-Tat 1–45 interaction (Additional file 3: Figure S3).

Then we wondered whether Tat protein was able to bind and to localize with TLR4 at the cell surface of HEK cells stably transfected with TLR4-MD2-CD14. Non-transfected HEK cells, HEK Null, were used as negative controls. To this end, Tat-TLR4-MD2 interaction was labelled with stained anti-Tat (Tat-555) and anti-TLR4 (TLR4-488) antibodies and complex formation was analyzed by confocal microscopy (Figure 3). Briefly, cells were incubated or not with Tat, and then labelled with anti-Tat or anti-TLR4 antibodies, separately or in a mixture. The results showed that Tat protein and its N-terminal fragment Tat 1–45 were able to bind to HEK-TLR4-MD2-CD14 cells but not to HEK Null (Figure 3). This labeling was specific since no staining was observed when experiments were performed after: i) incubating cells with the same amount of soluble GST protein instead of Tat (Figure 3), ii) omitting the primary antibody or iii) using an isotype control instead of anti-Tat antibodies (data not shown). This co-presence is also in agreement with the ability of Tat to interact physically with TLR4-MD2 in the inhibition and the biochemical binding assays previously described.

### Tat protein fails to stimulate TNF-α and IL-10 in macrophages from TLR4^−/−^ mice

**Figure 4:**
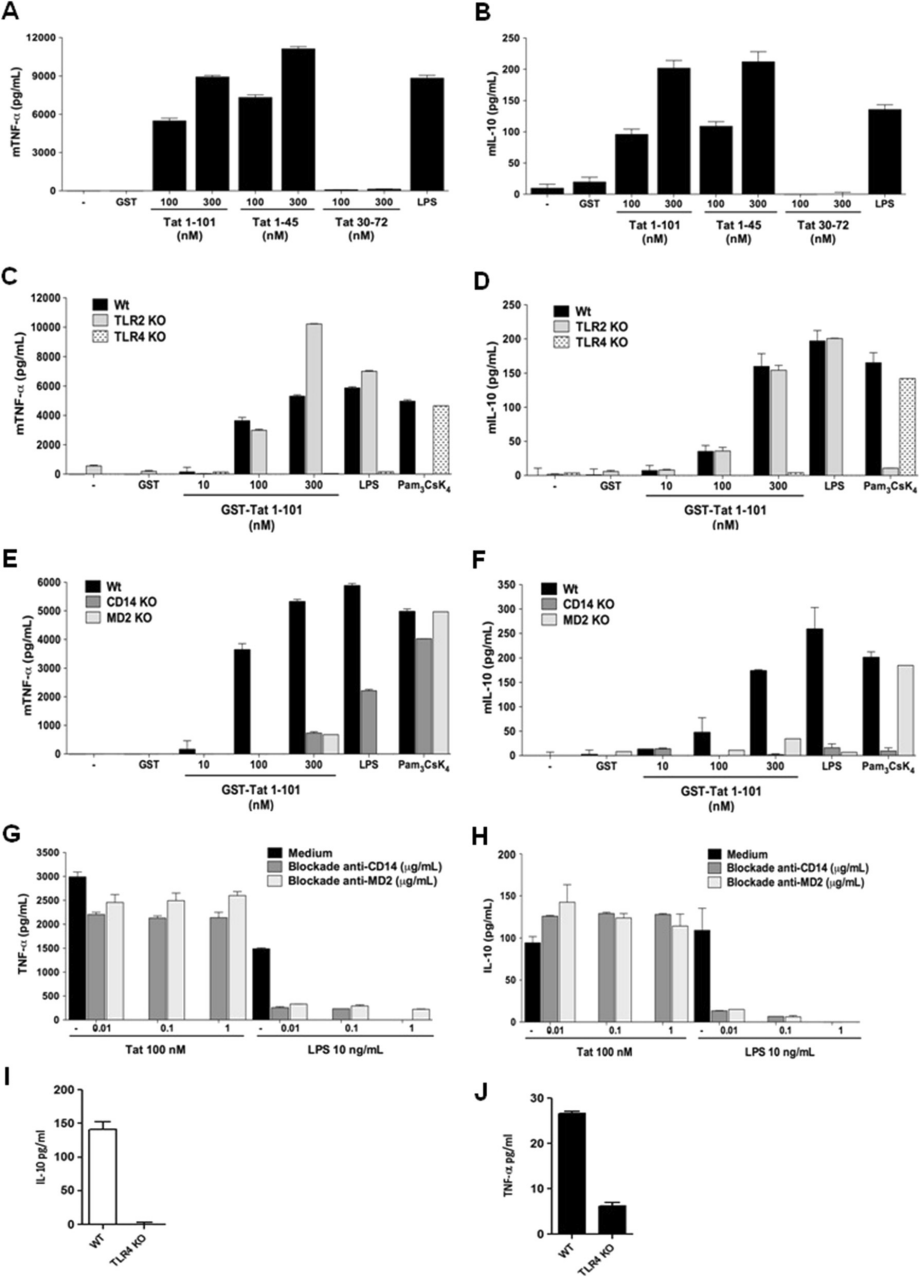
**Tat protein fails to stimulate TNF-α and IL-10 in macrophages from TLR4**^**−/− **^**MD2**^**−/−**^**, and CD14**^**−/− **^**mice. A-B)** Tat induces mTNFα and mIL-10 production in wt C57Bl/6 murine macrophages**.** Peritoneal macrophages (5.10^5^/well) from wild type C57BL/6 mice were stimulated for 24 h with increasing concentration of GST-Tat 1–101, GST-Tat 1–45, GST-Tat 30–72 or GST as control. LPS was used as a positive control. **C-F)** TLR4, CD14 and MD2 cell surface membrane proteins are essential for TNF-α and IL-10 production by Tat. Macrophages were isolated from wt mice or mice KO for TLR4, TLR2 **(C-D)** or CD14 or MD2 **(E-F)**. The cells were stimulated with increasing concentrations of GST-Tat 1–101, GST-Tat 1–45 or GST as control. Positive control experiments were performed by using the following TLR ligands: LPS (TLR4-CD14-MD2) and Pam_3_CsK_4_ (TLR2-CD14). **G-H)** Human monocytes were pretreated with blocking antibodies, anti-CD14 or anti-MD2 (0.1 - 1 μg/ml) for 60 min. Cells were then stimulated with GST or GST-Tat 1–101 (100 nM). **I-J)** TNF-α and IL-10 production in peritoneal washes from wt **(I)** or TLR4 KO mice **(J)** injected with Tat protein. The data represent means +/− SD of three independent experiments.

To confirm the involvement of Tat-TLR4 interaction in the signalling pathways leading to cytokine production, we used genetically engineered mice deficient in various TLR or their cofactors, including MD2 and CD14. Firstly, we validated the ability of Tat protein to stimulate the production of TNF-α and IL-10 in peritoneal macrophages. Our results showed that Tat protein and its N-terminal Tat 1–45, but not Tat 30–72, stimulated specifically and in a dose dependent manner TNF-α and IL-10 production in murine wt macrophages (Figure 4A-B). In agreement with the implication of TLR4-MD2, we showed that, when murine macrophages from TLR4^−/−^ mice were stimulated in the same conditions, no production of TNF-α and IL-10 was observed (Figure 4C-D). Similar results were obtained with macrophages from C3H/HeJ mice, which have a missense mutation in the third exon of TLR4 (Additional file 4: Figure S4). In accordance with the selective involvement of TLR4, our results showed that Tat protein and Tat 1–45, continued to stimulate TNF-α and IL-10 production in macrophages from mice deficient for TLR2^−/−^ TLR3^−/−^, TLR7^−/−^ or TLR9^−/−^ (data not shown). As a positive control we showed that TLR2 pathway was not altered in macrophages obtained from TLR4 KO mice as shown by cytokines production following stimulation with Pam_3_CsK_4_ ligand (Figure 4C-D). Most interestingly, *in vivo* data, showed that intraperitoneal administration of Tat protein leads to the production of TNF-α and IL-10 in the peritoneal washes of wt mice whereas these cytokines were greatly reduced by 75% for TNF-α and remained undetectable for IL-10, in TLR4 KO mice ( Figure 4I-J).

Considering the role of MD2 in the interaction with Tat protein, we evaluated the importance of the *in vivo* expression of this cofactor in the induction of the signalling pathways leading to Tat-induced cytokine production. Using macrophages from MD2^−/−^ mice, we showed that deficiency in MD2 abolished the ability of Tat to induce the production of both TNF-α and IL-10 (Figure 4E-F). Using the same approach, the implication of CD14 was also evaluated by using macrophages obtained from CD14^−/−^ mice. Unexpectedly, despite the absence of direct Tat-CD14 interaction (Figure 2A-B), the presence of CD14 expression seems to be essential for the activation of TLR4-MD2 signalling pathway by Tat as shown by the absence of cytokine production (Figure 4E-F). However, these data seem to be in apparent contradiction with those obtained with blockade anti-MD2 and anti-CD14 antibodies, which were unable to block Tat-induced TNF-α and IL-10 production (Figure G-H). As controls, and in agreement with previously reported data, the same antibodies completely blocked LPS-induced cytokine production (Figure 4G-H). We also confirmed that stimulation with LPS at relatively high concentrations restored cytokine production in macrophages from CD14 deficient mice (data not shown).

Altogether, our data confirm the essential implication of TLR4 and its cofactors CD14 and MD2 in HIV-1 Tat signalling for the production of IL-10 and TNF-α in monocytes/macrophages.

## Discussion

Several reports have shown that Tat protein is able to bind to various cell membrane receptors [35]. However Tat-TLR4 interaction has not been reported previously. Numerous arguments allowed us to test this hypothesis: i) TLR4 is expressed by human monocytes, ii) TLR4 activation induces the production of pro-inflammatory and anti-inflammatory cytokines including TNF-α and IL-10, by activating MAPkinases, PKC and NF-κB pathways that we have previously demonstrated to be activated by Tat in primary human monocytes [11], iii) TLR4 have been reported, in addition to LPS, to interact with several other ligands including viral proteins [34].

In agreement with this hypothesis, our results showed that Tat-induced TNF-α and IL-10 production was strongly inhibited in the presence of anti-TLR4 blocking antibody.

In order to be expressed at the cell surface, and functional, TLR4 requires the action of several factors including MD2 and CD14, which form complexes at the cell membrane. Analysis of Tat interaction with TLR4-MD2, MD2 and CD14, by complementary approaches, showed that Tat protein was able to interact with high affinity, with TLR4-MD2 and MD2 but not with CD14. This binding was totally inhibited, in a dose dependent manner, with soluble TLR4-MD2 or MD2, thus demonstrating the specificity of these interactions. This conclusion is also in line with confocal microscopy analysis data, which showed the co-detection of Tat and TLR4 only in TLR4-MD2 expressing cells. Comparison of the dissociation constant values K_0.5_ of Tat-TLR4-MD2 (10^-9^ M) and Tat-MD2 (4.10^-9^ M), showed that K_0.5_ of Tat-TLR4-MD2 was 2.5 times smaller than that of Tat-MD2. This higher affinity of Tat for TLR4-MD2 complex may be due to a better stabilization of Tat interactions with the TLR4-MD2 complex than with the MD2 alone. On the other hand, it has been shown that LPS recognizes MD2 with a dissociation constant of about 2.3 10^-6^ M [36] a K_0.5_ value that is 500 times higher than that found for Tat-MD2. In addition, while a direct interaction between LPS and CD14 has been described in several reports [36-38], in our study, no detectable interaction was found between Tat and CD14 neither in the solid phase nor in pull-down binding assays.

At functional level and in agreement with our biochemical data, we showed that Tat protein and its N-terminal fragment Tat 1–45 induced the production of TNF-α and IL-10 in macrophages from wild type mice but not in macrophages from mice genetically deficient for TLR4, MD2 or CD14. While the importance of cell surface expression of TLR4 and MD2 seems to be in line with our biochemical data, results obtained with CD14 KO mice seem to be in apparent contradiction if we consider its inability to interact with Tat protein. This apparent contradiction is amplified by the fact that anti-CD14 antibodies, which continue to inhibit LPS activation, fail to inhibit Tat-induced cytokine production. This apparent discrepancy may be related to the importance of CD14 in the expression of a biologically active TLR4 or its recruitment at cholesterol rich domains corresponding to the signalling platform [34]. Also, it is interesting to note that anti-MD2 antibodies were able to block cytokine production by LPS, while these same antibodies failed to inhibit Tat-induced cytokines. These results, in association with those obtained with MD2 KO mice, also underline the role of MD2 in the trafficking and surface localization of TLR4 as previously reported [39]. In addition the capacity of LPS-RS, an antagonist known for its capacity to alter MD2-TLR4 signalling [33], to inhibit Tat-induced cytokines is also an additional argument for the recruitment of this signalling pathway by HIV-1 Tat protein.

Considering the crucial role of PRR in the anti-viral immune defense, some viruses have evolved multiple mechanisms to hijack the initial function of TLR to their advantage so as to escape the control of the immune system or to infect their targets. For example, the respiratory syncytial virus (RSV) by its F protein, activates TLR4 to induce pro-inflammatory response that is implicated in the rapid viral clearence [40,41]. Interestingly, the rate of viral clearence was significantly reduced in RSV infected TLR4-deficient mice [40,42]. Similarly, MMTV envelope glycoprotein also triggers TLR4 pathway to activate in one hand, B-cells, the major target cells of the virus, and on the other hand to induce the expression of the immunosupressive cytokine IL-10 [43], thereby establishing an immunosuppression state favorable for both the inhibition of anti-viral immune response and viral replication [44]. So, unlike the antiviral role of TLR4 in the clearence of RSV [40,42], MMTV and HIV-1 are able to hijack TLR4 pathway to induce the production of IL-10, which contribute in association with other immunosuppressive factors, as PD-1, PD-L1 and IDO (indoleamine 2,3 dioxygenase) [45,46] to divert efficient immune response and to the establishment of persistent infections. In addition, several studies have shown that in some conditions, IL-10 can synergise with inflammatory cytokines to enhance HIV-1 replication [47,48].

Interestingly, other viruses have developed strategies to interfere with TLR pathway activation. For example, vaccinia virus, by its A46R protein which shares a TIR-like domain, interferes with the TLR pathway to block both MyD88 dependent and independent signalling [49]. Similarly, hepatitis C virus by its NS5A protein, forms insoluble complexes with MyD88 and inhibits the activation of the TLR4 pathway in murine macrophages [50]. Measles virus by its hemagglutinin, interacts with TLR2 on human monocytes and ativates the expression of CD150, that is its own entry receptor. These reports indicate that TLR can be hijacked by endogenous or viral ligands to promote the establishment of a pathological state or to escape viral containment.

## Conclusions

Taken together, our results give the first description of a direct, high affinity interaction between HIV-1 Tat protein and TLR4/MD2. By hijacking this pathway, HIV-1, *via* its early expressed Tat protein, contributes to the establishment of an abnormal hyper-activation of the immune system *via* TNF-α and to the development of an immunosuppression state by the production of IL-10, a highly immunosuppressive cytokine.

The understanding of the molecular and cellular mechanisms by which HIV Tat protein hijack the TLR4-CD14-MD2 receptor pathway represents a crucial interest for understanding the mechanisms recruited by HIV-1 to induce immunosuppression and for the development of new therapeutic strategies for future treatments.

## Methods

### Monocyte isolation

PBMCs were isolated from buffy coat of healthy HIV-1-negative donors by Ficoll density gradient. Briefly, PBMC were counted and resuspended in a 60/30 complete medium (60% AIMV, 30% Iscove; (Gibco)) containing 1% foetal calf serum (FCS), penicillin (100 IU/mL) and streptomycin (100 μg/ml). Monocytes were separated from lymphocytes by adherence to tissue culture plastic (Beckton Dickinson). After an incubation of 1 h at 37°C 5% CO_2_, non-adherent cells were removed and adherent cells (> 94% CD14^+^ by flow cytometric analysis) were washed and cultured in 60/30 complete medium containing 10% FCS, penicillin (100 IU/ml) and streptomycin (100 μg/ml) before being used in the experiments.

### Human embryonic kidney 293 cell line

Transfected HEK cell line stably expressing TLR4, TLR4-CD14-MD2, TLR2-CD14 and non-transfected HEK cell line (HEK Null) were purchased from Invivogen. HEK Null and HEK-TLR4 cell lines were cultured in DMEM supplemented with 10% FCS, normocin (100 μg/mL) and blasticidin (10 μg/ml); while HEK TLR4-CD14-MD2 and HEK TLR2-CD14 cell lines were grown in DMEM 10% FCS, normocin (100 μg/ml), blasticidin (10 μg/ml) and hygrogold (50 μg/ml) at 37°C and 5% CO_2._

### Primary mouse peritoneal macrophages and peritoneal washes

C57BL/6 were purchased from Charles Rivers. C3H/HeN and C3H/HeJ or Knockout (KO) mice C57BL/6: TLR4^−/−^, TLR2^−/−^, TLR3^−/−^, TLR9^−/−^, CD14^−/−^, MD2^−/−^ were obtained from (CNRS, Orléans, France). This study was conducted in accordance with the EU regulations and with the French national chart for ethics of animal experiments (articles R214-87 to 90 of the code rural). The protocol was approved by the committee on the ethics animal experiments of the Region Midi Pyrenée and by IFR 150 (permit numbers: 04-U563-DG-06 and MP/18/26/04/04). To minimize suffering, all animals were handeled under anesthezia. Primary macrophages were isolated as previously described [51]. Briefly, mice were injected intra peritoneally with 1 ml of thioglycolate medium 3% (Biomerieux). Three days later, the mice were sacrificed and macrophages were recovered by peritoneal washes and then enriched by adherence selection for 1 h in complete medium (DMEM supplemented with or without 2% FCS, penicillin (100 IU/ml) and streptomycin (100 μg/ml). Isolated macrophages were characterized by FACS analysis for the expression of CD11b^+^. Primary macrophages and peritoneal washes were obtained from two groups of mice. Wt (3 animals) and TLR4 KO (3 animals) mice were injected intraperitoneally with 100 μg of Gst-Tat at days 0, 1 and 3. 24 h after the last injection peritoneum was washed with 5ml of PBS and cytokines were quantified.

### Tat protein and TLR ligands

Recombinant HIV-1 Tat protein1-86 was obtained from “Agence Nationale de la Recherche sur le SIDA” (Paris, France). Synthetic Tat was obtained from E Loret (CNRS Marseille). Recombinant GST and deleted Tat mutant proteins were produced in our laboratory as previously described [11]. The level of endotoxin was assessed using the LAL assay (Bio-Sepra, France). All these recombinant proteins contained less than 0.3 EU/μg, the limit of detection of this test. LPS from E. *coli*, serotype R515 was purchased from Alexis biochemicals. Pam3CsK4 and LPS-RS from *R Sphaeroides*, were obtained from InvivoGen (San Diego, CA). Human IFN-γ was purchased from eBioscience.

### Antibodies and chemical inhibitors

For inhibition experiments: anti-human TLR4 (clone HTA125), anti-TLR2 (clone TL2.1), anti-CD14 (clone 61D3) and mouse IgG2a isotype were obtained from eBioscience. Anti-CD14 (clone RMO52 and clone MY4) and mouse IgG isotype controls were from Beckman Coulter; anti-MD2 (clone 288307) was from R&D Systems. Mab anti-Tat antibodies were obtained from ANRS (Paris). For confocal imaging and flow cytometry analysis: goat anti-hTLR4 was from R&D System. Unlabelled mouse anti-hTLR4 and labelled mouse anti-hTLR4-PE were from eBioscience. Alexa 488 or 555 labelled IgG against rabbit and goat IgG respectively, were from Invitrogen. For western blot analysis, recombinant hMD2, rhCD14 and rhTLR4-MD2, monoclonal anti-hMD2 and anti-hTLR4 were from R&D systems. Mabs CD14 (MEM-18) was from Santa Cruz Biotechnology.

### Cytokine detection by ELISA

Adherent monocytes (10^6^/well), murine macrophages (5.10^5^/well) or HEK cells (5.10^5^/well) were washed 3 times with cold PBS. Cells were then cultured in the presence of 1% FCS. After 24 h of cell treatment, the supernatants were collected and analyzed for human and mouse TNF-α and IL-10. Cytokine amounts were determined using ELISA kits from BD Biosciences and R&D Systems according to the manufacturers’ instructions.

### Confocal microscopy

HEK cells were grown on 12-mm round coverslides at 60–80 % confluence. They were then incubated with GST-Tat (100 nM) for 15 min. After stimulation and washing with PBS, cells were fixed with 4% paraformaldehyde-PBS for 10 min. After 3 washes, they were incubated with 50 mM of NH_4_Cl for quenching. This step saturated free aldehydes to inhibit auto-fluorescence. After extensive washing, the cells were saturated with PBS-BSA 5% for 30 min. For colocalization experiments, GST-Tat and TLR4 proteins were labelled for 45 min at room temperature with 10 μg/ml of mouse (Mab anti-Tat) or goat primary polyclonal anti TLR4 antibodies. After washes, Tat and TLR4 antibodies were labelled with the corresponding secondary antibodies: Alexa Fluor-488 or Alexa Fluor-555-conjugated antibodies directed against mouse or goat immunoglobulin G (1/500) for 45 min at room temperature. After three washes with PBS or PBS-MgCl_2_ 150 mM, cell nuclei were stained at room temperature with DAPI or chromomycin A3 in PBS-MgCl_2_ 150 mM for 1h. The images were taken with a confocal microscope (Zeiss Axiomager LSM 710 META scanning unit and a 1.4NA × 63 objective). Cellular localization were analyzed and processed with ImageJ.

### GST pull-down and Co-immunoprecipitation assays

For GST pull-down, equal amounts of GST, GST-Tat 1–45, GST-Tat 30–72 or GST-Tat 1–101 proteins coupled to glutathione agarose beads were saturated with BSA (250 μg/ml) for 2 h at 4°C. After washing (Tris–HCl 20 mM, NaCl 150 mM), agarose fixed proteins were incubated with 1 μg of TLR4-MD2, MD2 or CD14 soluble recombinant human proteins (R&D Systems) or total cellular extracts (500 μg) from HEK 293 or HEK 293-TLR4-MD2-CD14 cells. The beads were then washed extensively with Tris–HCl 20 mM, NaCl 150 mM, NP-40 0.5%, PMSF 0.5 mM, leupeptin 10 μg/mL, Na_3_VO_4_ 0.2 mM, NaF 0.05 mM and the presence of retained TLR4, MD2 or CD14 proteins was analyzed by SDS-PAGE and western blot using specific antibodies. Recombinant human MD2, TLR4-MD2, TLR4 or CD14 were coated at the indicated concentrations in 96 well plates for 24 h at 4°C. After washing with PBS-0.05% Tween20, wells were saturated with PBS-0.05% Tween 20, 5% non-fat milk for 1 h at 37°C. After 3 washes, various amounts of GST, GST-Tat 1–101 or Tat deleted mutants were incubated for 2 h at 37°C. For competition assays, GST-Tat or its deleted mutants previously incubated for 1 h at 37°C with different ligands were added to compete with the coated proteins (MD2 or TLR4-MD2) in the wells for an additional 2 h. After washing, binding was detected by anti-GST (1/500) or monoclonal anti-Tat antibodies (10 μg/ml) as described [9].

### Statistical tests

All statistical analyses used the Student’s t-test, unpaired for normal distribution, for at least three independent experiments. Differences were considered significant at p values < 0.05. Microsoft Excel and Prism were used to construct the plots and measure means, standard deviations and p values.

## Competing interests

The authors declare that they have no competing interests.

## Authors’ contributions

NB, KL, RP, NT and EB conceived and designed the experiments. NB, KL, and RP performed the experiments. NB, KL, RP, and EB analyzed the data. EB and NB wrote the paper. All authors read and approved the final manuscript.

## Supplementary Material

Additional file 1: Figure S1 LPS-induced TNF-α and IL-10 is TLR4-dependent. Monocytes were pretreated or not with increasing amounts of blocking antibodies against TLR4 or TLR2 or isotype control for 1h before stimulation by LPS 1 ng/mL. TNF-α and IL-10 production were quantified in the culture supernatants by ELISA. Data represent means +/− SD (n=3). Click here for file

Additional file 2: Figure S2 Characterization of recombinant proteins. A) Equal amounts (1μg) of recombinant GST-Tat proteins were separated by SDS-PAGE at 10% and stained by coomassie blue dye. B) recombinant GST-Tat proteins analysis by western blot. Proteins were labeled by using a monoclonal anti-Tat directed against the N-terminal region 1–15. C) Recombinant TLR4, TLR4-MD2, MD2, CD14 proteins were separated by SDS-PAGE at 10% and stained by coomassie blue dye. Click here for file

Additional file 3: Figure S3 Tat interacts specifically and with high affinity with MD2 and TLR4-MD2. A-B) Increasing amounts of rhMD2 were coated in the wells. After incubation with a constant amount of GST-Tat 1–101 or GST-Tat 1–45 (1 μM), the binding of Tat to rhMD2 was detected by using anti-GST antibodies (1/500). The data represent OD at 450 nm +/− SD (triplicate) and are representative of one of three independent experiments. C-D) Increasing concentrations of GST-Tat 1–101 or GST-Tat 1–45 were incubated for 2 h with 10^-2^ μg/mL of coated rhMD2. The data represent OD at 450 nm +/− SD (triplicate) and are representative of one of three independent experiments. E) rhMD2 and rhTLR4-MD2 compete for Tat-rhMD2 interaction: GST-Tat 1–101 and 1–45 (0.1 μM) were pre-incubated for 1 h with PBS (control) or with increasing amounts of soluble rhTLR4-MD2, rhTLR4 or rhCD14 before incubation with the coated rhMD2. Binding of Tat to rhMD2 was analyzed as described above by measuring OD at 450 nm. Data represent mean +/− SD (n ^3^3). Click here for file

Additional file 4: Figure S4 Tat protein fails to stimulate TNF-α and IL-10 in macrophages from TLR4^−/−^ MD2^−/−^, and CD14^−/−^ mice. Macrophages were isolated from Wt mice (C3H/HeN) or mice deficient for TLR4 signalling (C3H/HeJ). The cells were stimulated with increasing concentrations of Tat 1–86, GST-Tat 1–101, GST-Tat 1–45, GST as control or LPS. Mouse TNF-α and IL-10 production were determined by ELISA. Data representative of three independent experiments (mean +/− SD). Click here for file
