# Supplementary figures and images for: HIV-1 Tat protein binds to TLR4-MD2 and signals to induce TNF-α and IL-10
Source: Retrovirology. 2013 Oct 28;10:123. doi: 10.1186/1742-4690-10-123 (PMC4231456; doi:10.1186/1742-4690-10-123)

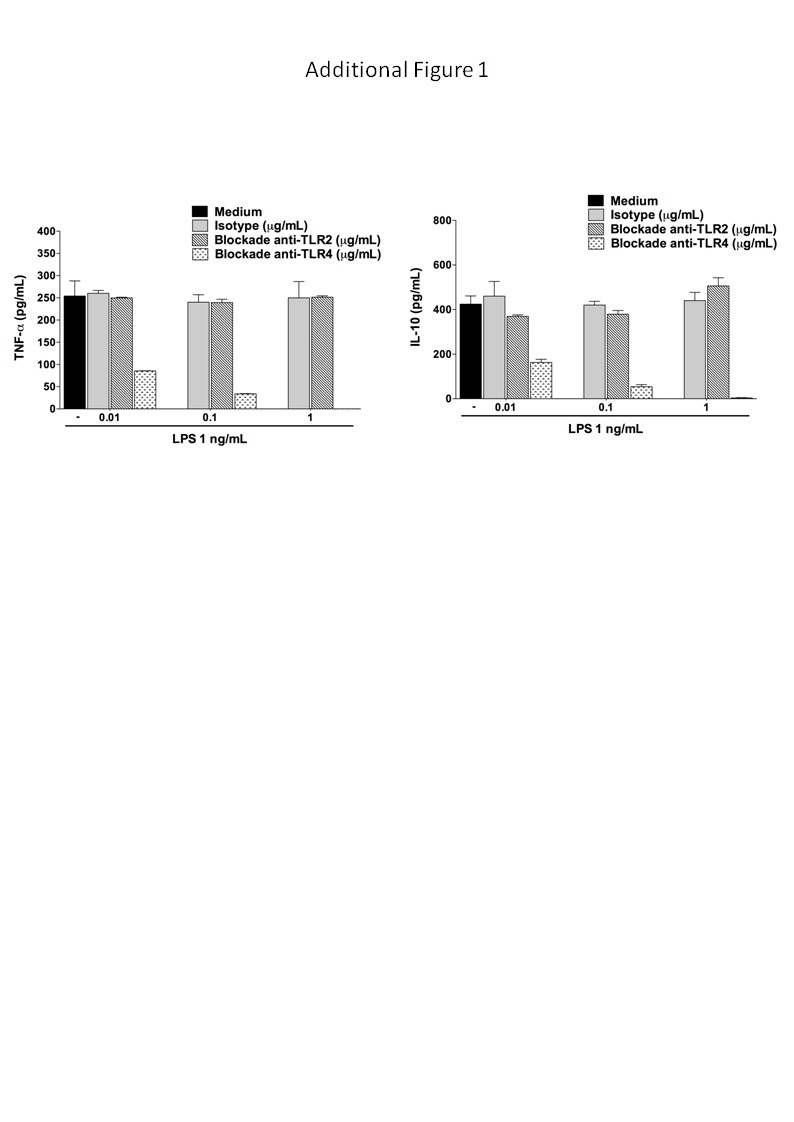

Supplement: Additional file 1: Figure S1 — LPS-induced TNF-α and IL-10 is TLR4-dependent. Monocytes were pretreated or not with increasing amounts of blocking antibodies against TLR4 or TLR2 or isotype control for 1h before stimulation by LPS 1 ng/mL. TNF-α and IL-10 production were quantified in the culture supernatants by ELISA. Data represent means +/− SD (n=3). [file 1742-4690-10-123-S1.jpeg]

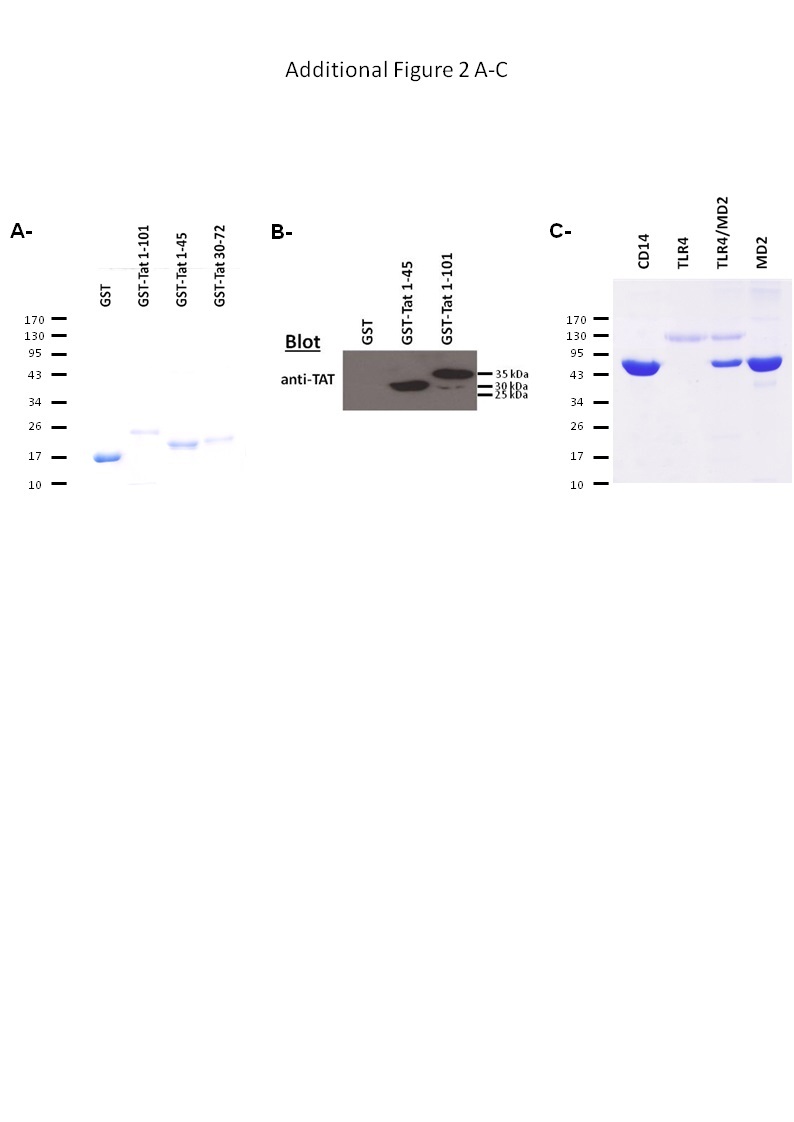

Supplement: Additional file 2: Figure S2 — Characterization of recombinant proteins. A) Equal amounts (1μg) of recombinant GST-Tat proteins were separated by SDS-PAGE at 10% and stained by coomassie blue dye. B) recombinant GST-Tat proteins analysis by western blot. Proteins were labeled by using a monoclonal anti-Tat directed against the N-terminal region 1–15. C) Recombinant TLR4, TLR4-MD2, MD2, CD14 proteins were separated by SDS-PAGE at 10% and stained by coomassie blue dye. [file 1742-4690-10-123-S2.jpeg]

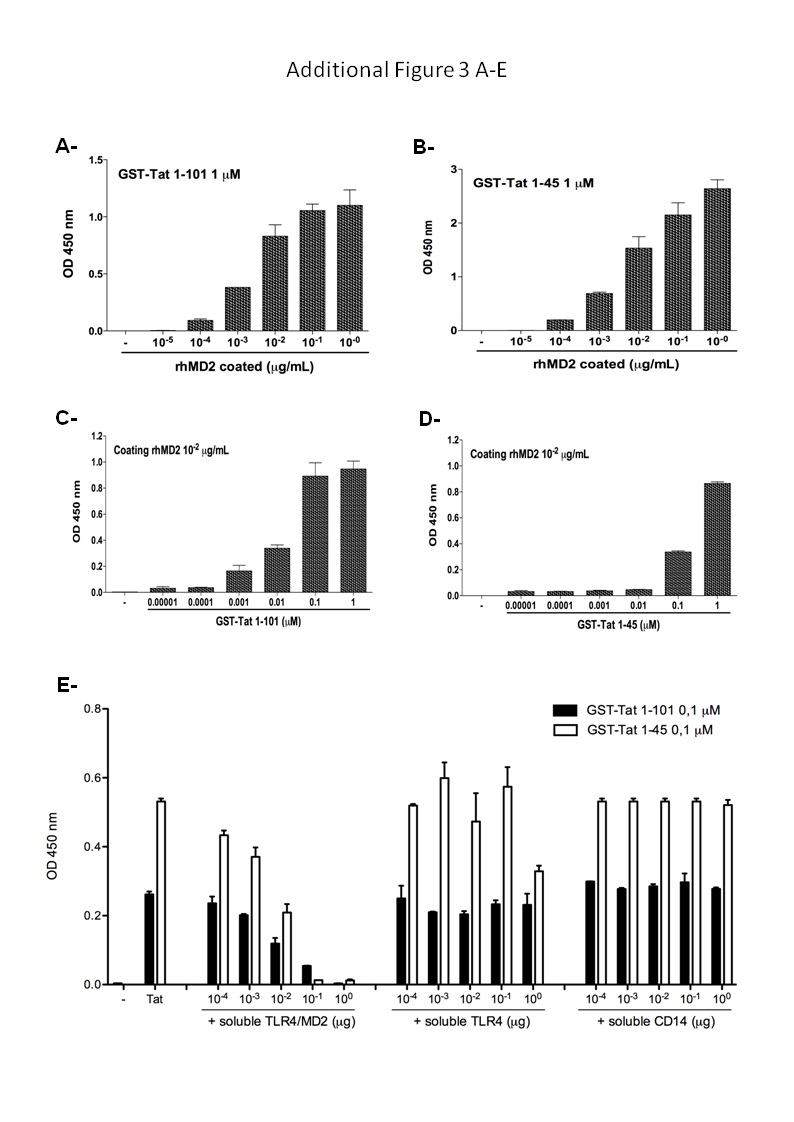

Supplement: Additional file 3: Figure S3 — Tat interacts specifically and with high affinity with MD2 and TLR4-MD2. A-B) Increasing amounts of rhMD2 were coated in the wells. After incubation with a constant amount of GST-Tat 1–101 or GST-Tat 1–45 (1 μM), the binding of Tat to rhMD2 was detected by using anti-GST antibodies (1/500). The data represent OD at 450 nm +/− SD (triplicate) and are representative of one of three independent experiments. C-D) Increasing concentrations of GST-Tat 1–101 or GST-Tat 1–45 were incubated for 2 h with 10-2 μg/mL of coated rhMD2. The data represent OD at 450 nm +/− SD (triplicate) and are representative of one of three independent experiments. E) rhMD2 and rhTLR4-MD2 compete for Tat-rhMD2 interaction: GST-Tat 1–101 and 1–45 (0.1 μM) were pre-incubated for 1 h with PBS (control) or with increasing amounts of soluble rhTLR4-MD2, rhTLR4 or rhCD14 before incubation with the coated rhMD2. Binding of Tat to rhMD2 was analyzed as described above by measuring OD at 450 nm. Data represent mean +/− SD (n 33). [file 1742-4690-10-123-S3.jpeg]

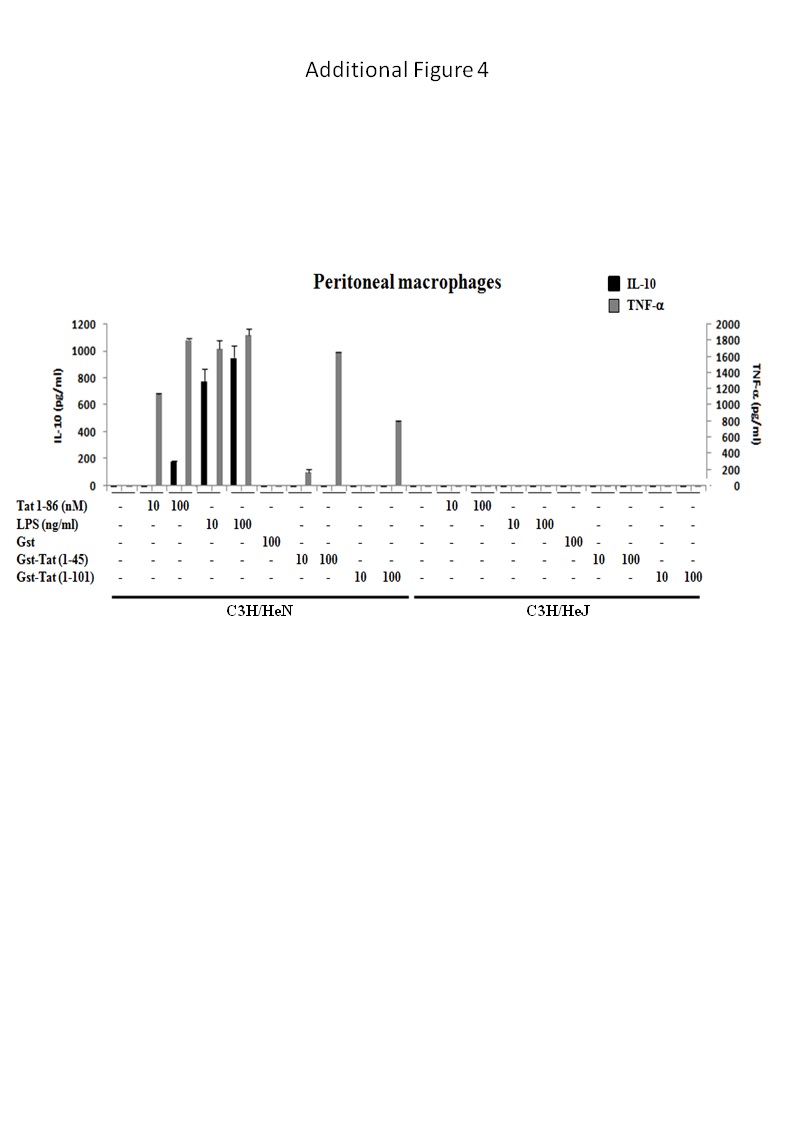

Supplement: Additional file 4: Figure S4 — Tat protein fails to stimulate TNF-α and IL-10 in macrophages from TLR4−/− MD2−/−, and CD14−/− mice. Macrophages were isolated from Wt mice (C3H/HeN) or mice deficient for TLR4 signalling (C3H/HeJ). The cells were stimulated with increasing concentrations of Tat 1–86, GST-Tat 1–101, GST-Tat 1–45, GST as control or LPS. Mouse TNF-α and IL-10 production were determined by ELISA. Data representative of three independent experiments (mean +/− SD). [file 1742-4690-10-123-S4.jpeg]
